# MicroRNA Signature of Human Microvascular Endothelium Infected with *Rickettsia rickettsii*

**DOI:** 10.3390/ijms18071471

**Published:** 2017-07-09

**Authors:** Abha Sahni, Hema P. Narra, Jignesh Patel, Sanjeev K. Sahni

**Affiliations:** Department of Pathology, University of Texas Medical Branch, Galveston, TX 77555, USA; hpnarra@utmb.edu (H.P.N.); jgpatel@utmb.edu (J.P.)

**Keywords:** microRNAs, microarray, endothelial cells, *Rickettsia rickettsii*, *NOTCH1*

## Abstract

MicroRNAs (miRNAs) mediate gene silencing by destabilization and/or translational repression of target mRNA. Infection of human microvascular endothelial cells as primary targets of *Rickettsia* *rickettsii*, the etiologic agent of Rocky Mountain spotted fever, triggers host responses appertaining to alterations in cellular gene expression. Microarray-based profiling of endothelial cells infected with *R.* *rickettsii* for 3 or 24 h revealed differential expression of 33 miRNAs, of which miRNAs129-5p, 200a-3p, 297, 200b-3p, and 595 were identified as the top five up-regulated miRNAs (5 to 20-fold, *p* ≤ 0.01) and miRNAs 301b-3p, 548a-3p, and 377-3p were down-regulated (2 to 3-fold, *p* ≤ 0.01). Changes in the expression of selected miRNAs were confirmed by q-RT-PCR in both in vitro and in vivo models of infection. As potential targets, expression of genes encoding *NOTCH1*, *SMAD2*, *SMAD3*, *RIN2*, *SOD1*, and *SOD2* was either positively or negatively regulated. Using a miRNA-specific mimic or inhibitor, *NOTCH1* was determined to be a target of miRNA 200a-3p in *R. rickettsii*-infected human dermal microvascular endothelial cells (HMECs). Predictive interactome mapping suggested the potential for miRNA-mediated modulation of regulatory gene networks underlying important host cell signaling pathways. This first demonstration of altered endothelial miRNA expression provides new insights into regulatory elements governing mechanisms of host responses and pathogenesis during human rickettsial infections.

## 1. Introduction

*Rickettsia rickettsii* and *R. conorii* are arthropod-borne, Gram-negative, obligate intracellular bacterial pathogens and the causative agents of Rocky Mountain spotted fever (RMSF) and Mediterranean spotted fever (MSF) in humans. Capable of invading and multiplying within a variety of host cell types in vitro, pathogenic rickettsiae preferentially exploit microvascular endothelial cells of small and medium-sized blood vessels as their primary targets to gain access to the nutrient-rich cytoplasmic environment conducive to their growth, multiplication, spread, and dissemination in the mammalian hosts. Consequently, vasculitis attributed to endothelial activation, inflammation, and damage/dysfunction, and resultant perturbation of the microvasculature’s barrier function leading to fluid imbalance and edema of the lungs and brain as vital organ systems comprise the salient features of rickettsial pathogenesis. In that regard, transcriptional activation of endothelial cells infected with *R. rickettsii* and *R. conorii* displaying a pro-adhesive, pro-inflammatory, and pro-thrombotic phenotype is characterized by induced expression of surface adhesion molecules and tissue factor and enhanced expression and secretion of a number of cytokines and chemokines [1,2,3,4]. Also, stimulation of anti-oxidant defense mechanisms to counter the oxidative stress due to infection and activation of immediate early signal transduction pathways are now well understood [5,6,7].

MicroRNAs (miRNAs) expressed by eukaryotic cells are evolutionarily conserved, single-stranded, non-coding small RNAs that are typically 21–23 nucleotides long and function by targeting cellular messenger RNAs (mRNAs) for degradation and/or suppression of protein translation based on sequence complementarity between the miRNA and targeted mRNA. In recent years, miRNAs have been documented to play pivotal regulatory roles in diverse biological processes such as cellular development, differentiation, proliferation, and metabolism [8,9,10,11]. As novel post-transcriptional regulators of gene expression, miRNAs have also been implicated as important determinants of the dynamics of the immune response during normal and pathogenic immunological situations and disease biomarkers [12]. MicroRNAs are transcribed by RNA polymerase II and III, generating precursors that undergo a series of cleavages to form mature miRNA. In the cytoplasm, the miRNA guide strand associates with Argonaute (Ago) within the RNA-induced silencing complex (RISC), which serves as the primary mechanism for targeting the RISC complex to mRNAs. Because mRNA targets are usually recognized through complementary base pairing with a short, 2–7 nucleotides long seed sequence often present at the 5′ end of the miRNA, a single miRNA:RISC complex can silence hundreds of mRNAs [13]. Once bound by the RISC, miRNAs usually target the 3′-untranslated region (UTR) of mRNAs, resulting in the degradation of the target mRNA and/or repression of translation.

It is now established that about 30% of human genes are regulated post-transcriptionally by different miRNAs [14] and it is also becoming increasingly evident that miRNAs play significant roles in host-pathogen interactions as a critical regulatory control in the mammalian immune system. In this regard, an emerging theme is the identification of virally encoded miRNAs involved in the regulation of host and/or viral gene expression and mechanism of immunoevasion [15,16,17]. However, considerably less information is available about the effects of intracellular bacterial pathogens on host miRNA expression and the potential roles of host-derived miRNAs in modulating such infections via determination of host responses. Intracellular bacteria generally employ sophisticated strategies to preserve the host cell for their growth and replication and prevent their recognition by the innate immune system [18]. Therefore, an evaluation of miRNA-based regulatory mechanisms underlying the host cell responses to rickettsial infections is of critical importance to understand disease pathogenesis and to develop new strategies for improved diagnosis and therapeutics. The aim of this study was to investigate the miRNA profile of endothelial cells infected with *R. rickettsii* and to validate correlative changes in the expression of select miRNAs and their target genes.

## 2. Results

### 2.1. MicroRNA Expression Profiling of Microvascular Endothelial Cells Infected with R. rickettsii

To determine the extent of differential regulation of miRNAs, we initially performed human Affymetrix GeneChip^®^ miRNA microarray on RNA isolated from human microvascular endothelial cells infected with *R. rickettsii* at an MOI of 1:5 for 3 and 24 h and simultaneously processed uninfected controls. This expression profiling study led to the identification of 33 miRNAs that revealed consistent pattern of change in three independent samples and were significantly altered (cut-off fold-change of ≥2.0, *p* ≤ 0.05) in infected endothelial cells as compared to the corresponding mock-infected controls (Table 1). Among these, 23 cellular miRNAs were expressed at significantly higher levels in comparison to uninfected endothelial cells, while the expression levels of 10 miRNAs were decreased as a consequence of *R. rickettsii* infection. Our data further revealed that a total of 4 and 9 miRNAs’ expression increased at 3 and 24 h post-infection, respectively, whereas the remaining 10 displayed induced expression at both time points. A similar analysis of down-regulated miRNAs suggested reduced levels of expression of 2 miRNAs at 3 h and 5 miRNAs at 24 h, whereas 3 miRNAs were found to be expressed at significantly reduced levels at both 3 and 24 h post-infection. The identities and consensus sequences of miRNAs shown to be either up- or down-regulated in response to *R. rickettsii* infection of cultured human endothelial cells are listed in Table 1. These results from a global screening approach suggest that expression of a number of miRNAs is significantly altered by *R. rickettsii* infection of host endothelial cells.

### 2.2. Confirmatory Analysis of Differentially Regulated miRNAs Using In Vitro and In Vivo Models of Infection

**Figure 1:**
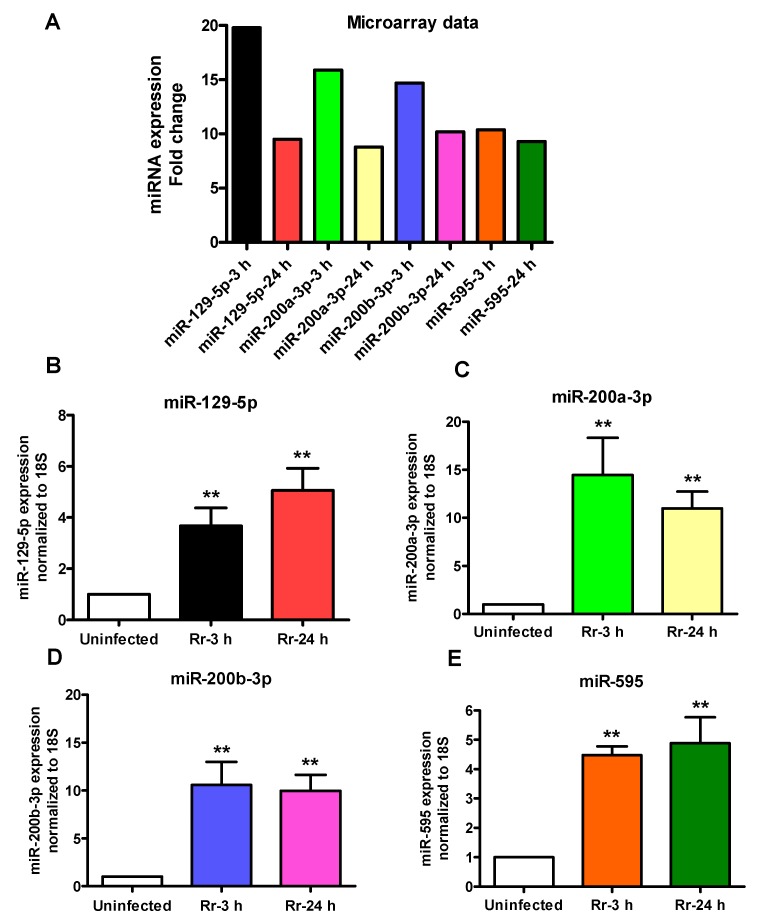
Expression of upregulated miRNAs in *R. rickettsii*-infected HMECs: HMECs were infected with *R. rickettsii* for 3 and 24 h, RNA was extracted, and microarray (**A**) or q-RT-PCR (B-E) were performed to measure the expression of selected miRNAs. The data is normalized to 18S rRNA and relative expression is calculated by ^ΔΔ^*C*_T_ method. The results are presented as the mean (**A**) or mean ± SE (**B**–**E**) of three independent experiments. The asterisks indicate statistically significant change (*p* < 0.01).

**Figure 2:**
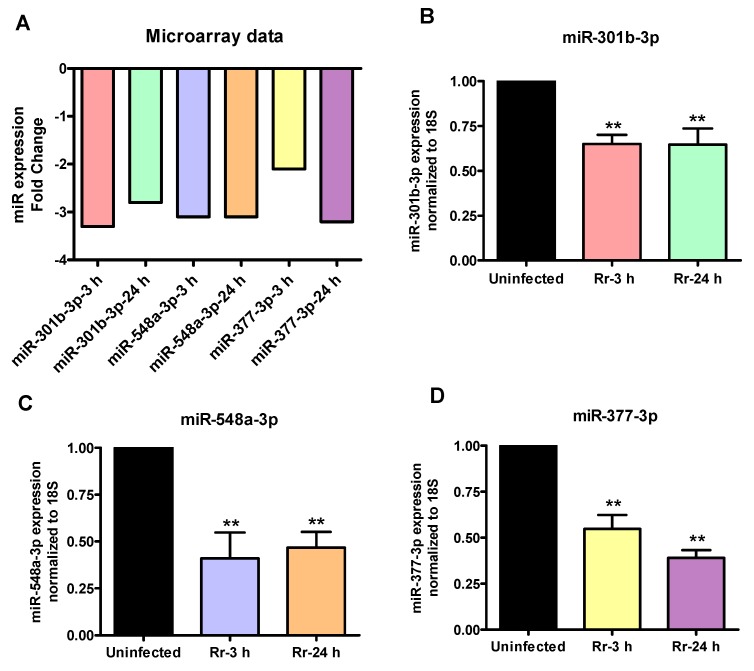
Expression of down-regulated miRNAs in *R. rickettsii*-infected HMECs: HMECs were infected with *R. rickettsii* for 3 and 24 h, RNA was extracted, and microarray (**A**) or q-RT-PCR (**B**–**D**) were performed to measure the expression of selected down regulated miRNAs. The data is normalized to 18S rRNA and relative expression is calculated by ^ΔΔ^*C*_T_ method. The results are presented as the mean (**A**) or mean ± SE (**B**–**D**) of three independent experiments. The asterisks indicate statistically significant change (*p* < 0.01).

To validate our microarray results, q-RT-PCR based Taqman miRNA assays were performed to determine the expression levels of four significantly up-regulated and three down-regulated miRNAs (Figure 1). These miRNAs were selected based on their cellular abundance and differential expression during the infection. Generally corresponding to the microarray results (Figure 1A), q-RT-PCR based analysis of miR-129-5p (B), miR-200a-3p (C), miR-200b-3p (D), and miR-595 (E) also revealed a pattern of significantly increased expression in infected host cells. However, the extent of up-regulation (the fold-change over basal expression in uninfected cells) for miR-129-5p and miR-595 at both 3 h and 24 h post-infection was considerably higher in our microarray data (Figure 1A). Similarly, q-RT-PCR based analysis of the expression patterns of miR-301b-3p, miR-548a-3p, and miR-377-3p, which were determined to display diminished expression by microarray-based measurements (Figure 2A), also demonstrated consistent, statistically significant down-regulation of all three miRNAs (Figure 2B–D). These findings thus indicate that changes in miRNA abundance identified by whole genome microarray accurately capture the steady-state miRNA contents of uninfected and infected cells and that expression patterns generated by application of both microarray and individual q-RT-PCR assays suggest consistent changes for all of the tested miRNAs, confirming their differential expression during *R. rickettsii* infection of cultured human endothelial cells.

**Figure 3:**
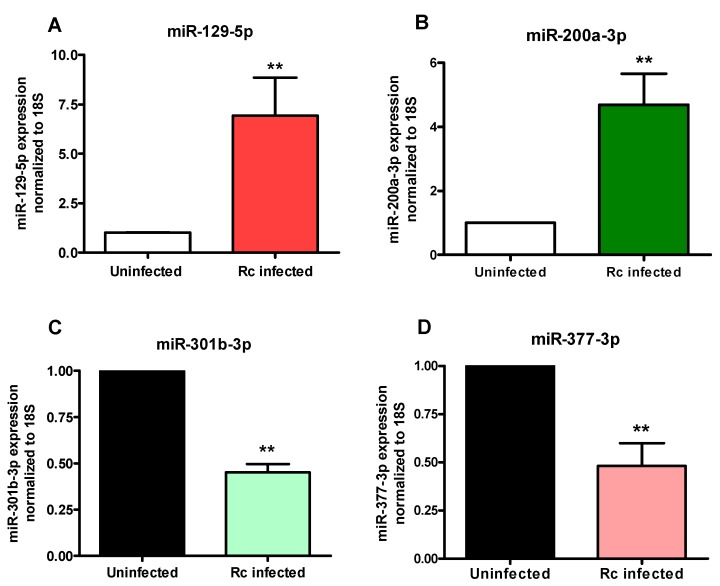
Expression of miRNAs in *R. conorii*-infected C_3_H/HeN mice in vivo: Mice were infected with *R. conorii* (2.25 × 10^5^ pfu) intravenously. Control animals received injection of saline. On day 3 post-infection, mice were euthanized, lungs were removed, RNA was isolated, and expression of miRNAs was measured by q-RT-PCR. Panels A through D illustrate the expression levels of miRNAs 129-5p, 200a-3p, 301b-3p, and 377-3p, respectively. The asterisks indicate statistically significant change (*p* < 0.01).

As an important corollary to these in vitro findings, we further analyzed the expression status of miR-129-5p, miR-200-3p, miR-301b-3p, and miR-377-3p in the lungs of mice infected with *R. conorii*, another spotted fever group pathogen phylogenetically and antigenically similar to *R. rickettsii. R. conorii* infection of susceptible mice closely recapitulates the pathogenesis of endothelial-target rickettsiosis in humans and is widely accepted as an established model for the investigations of regulation of gene expression and mechanisms of both innate and adaptive immunity during spotted fever rickettsioses [19]. Again, q-RT-PCR based quantitation on day 3 post-infection (Figure 3) revealed about 7.0 ± 1.9-fold higher expression of miR-129-5p (A) and 4.7 ± 0.9-fold up-regulation of miR-200a-3p (B) in the lungs of infected mice when compared to the uninfected controls and a decrease of about 53 ± 5% in the levels of miR-301b-3p (C) and 51 ± 10% in miR-377-3p (D), respectively. These results further ascertain changes in the expression of selected miRNAs in a mouse model of infection and indicate that in vivo changes in the expression patterns of selected miRNAs coincide with in vitro findings from cultured endothelial cells as the host.

### 2.3. Bioinformatics-Based Prediction of mRNA Targets for Differentially Regulated miRNAs

As important regulators of gene expression, miRNAs function by guiding the RNAi-induced silencing complex (RISC) to partially complementary sequences in target mRNAs to suppress gene expression by mRNA decay and/or inhibition of translation. Complete complementarity between a miRNA and its target mRNA is rare and a match of as little as 6 base pairs (seed region) is often sufficient to suppress gene expression. Accordingly, several miRNAs can target a single gene and a single miRNA can target multiple genes [20,21]. To identify the transcripts that may potentially be the targets of miRNAs in infected endothelial cells, we conducted computational searches of the miRTarBase and DIANA-TarBase, databases of experimentally validated miRNA-target interactions [22,23]. The experimentally validated gene targets for miRNAs undergoing either up- or down-regulation during *R. rickettsii* infection of host endothelial cells are listed in Table 2.

### 2.4. Correlation between Expression Profiles for miRNA-Target mRNA Pairs

**Figure 4:**
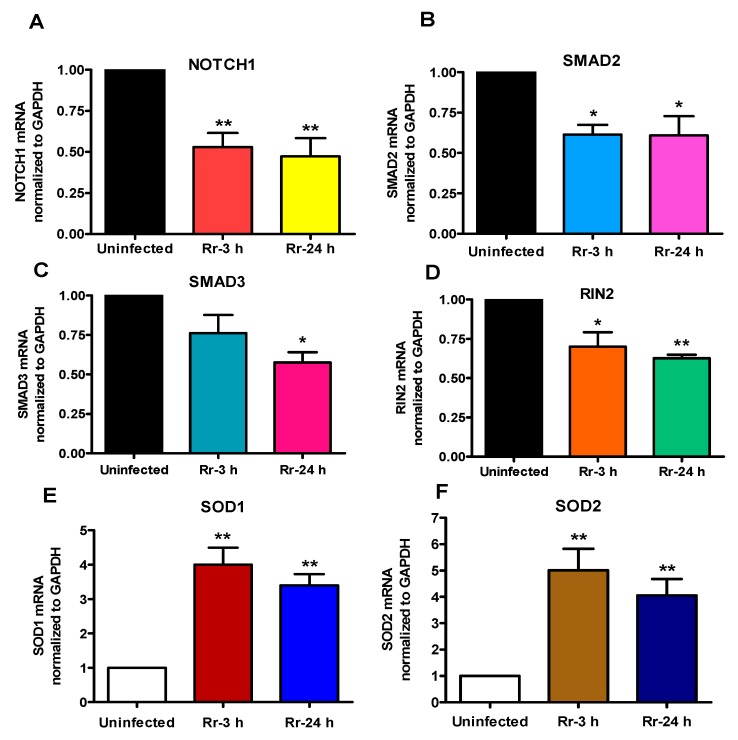
miRNA target genes expression during *R. rickettsii* infection of HMECs: miRNA target genes were identified as described above and selected target gene expression was measured during *R. rickettsii* infection of HMECs by q-RT-PCR using gene specific primers for *NOTCH1* (**A**), *SMAD2* (**B**), *SMAD3* (**C**) and *RIN2* (**D**), *SOD1* (**E**) and *SOD2* (**F**). The data presented as mean ± SE of three separate experiments. The asterisk (* = *p* < 0.05, ** = *p* < 0.01) indicates statistically significant change.

To investigate whether miRNA changes due to *R. rickettsii* infection correlate with the expression profiles of their target genes, we next determined the steady-state expression of selected genes (identified from Table 2). Figure 4 shows the relative expression of *NOTCH1* (A), *SMAD2* (B), *SMAD3* (C), and *RIN2* (D) mRNA as potential downstream targets of positively-regulated miRNAs miR-129-5p, miR-200a-3p, and miR-200b-3p. As expected, the findings demonstrate significant down-regulation of *NOTCH1*, *SMAD2*, and *RIN2* at 3 and 24 h following *R. rickettsii* infection, while the expression of *SMAD3* was decreased only at 24 h post-infection. We also quantified the expression of mRNAs for *SOD1* and *SOD2* as targets of miR-377-3p, and miR-301b-3p, both of which are subjected to negative regulation in *R. rickettsii*-infected host endothelial cells. Again, our results indicate significant induction of *SOD1* (E) and *SOD2* (F) mRNA post-infection in concordance with the decrease in miR-377-3p and miR-301b-3p expression (Figure 2). Thus, a ccombinatorial assessment of miRNA and gene expression patterns clearly suggests a correlation between miRNA and mRNA expression and the likelihood of the regulation of important host response genes by miRNAs.

### 2.5. miRNA200a-3p Controls NOTCH1 in R. rickettsii-Infected Endothelial Cells

**Figure 5:**
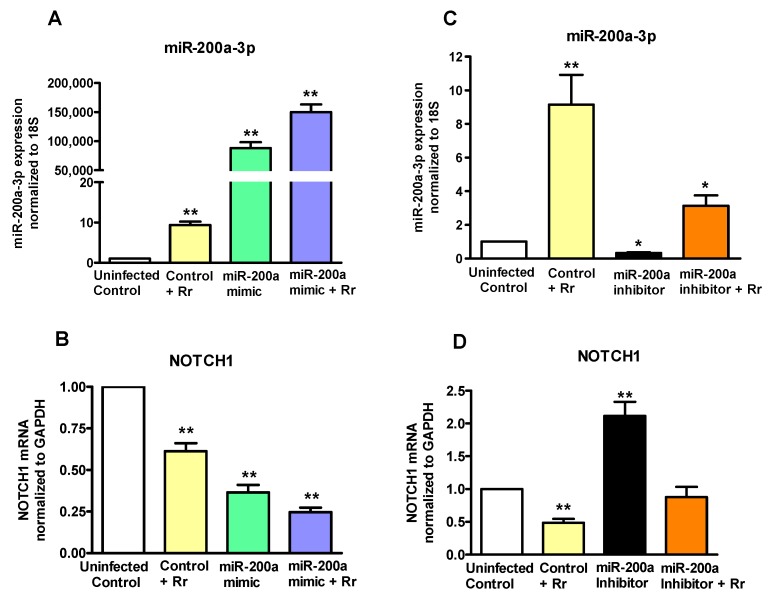
*NOTCH1* regulation by miRNA-200a-3p. HMECs were transfected with miRNA 200a-3p mimic (1 nM) (A and B) or inhibitor (200 nM) (**C** and **D**) for 24–48 h using Lipofectamine^®^ RNAiMAX according to the manufacturer’s instructions prior to infection with *R. rickettsii* for 24 h. Total RNA was isolated from cells lyzed in the Tri-reagent and miRNA 200a-3p (**A**,**C**) and *NOTCH1* (**B**,**D**) expression was measured by q-RT-PCR. The data presented as mean ± SE of three separate experiments. The asterisks (* = *p* < 0.05, ** = *p* < 0.01) indicate statistically significant change.

To investigate the possibility that miR-200a-3p may function as a regulator of *NOTCH1* during *R. rickettsii* infection, we designed experiments based on its gain- and loss-of-function in endothelial cells. A miR-200a-3p mimic, inhibitor, and the corresponding negative controls were delivered by transfection into endothelial cells and the resulting effects on the levels of miR-200a-3p and *NOTCH1* expression were monitored by q-RT-PCR. As expected, the mimic resulted in a dramatic increase in miR-200a-3p expression. Interestingly, infection with *R. rickettsii* resulted in further increase of miR-200a-3p expression in cells transfected with the mimic (Figure 5A). Conversely, introduction of miR-200a-3p inhibitor reduced the cellular miRNA levels by ≥60% and infection-induced miR-200a-3p expression was also decreased by about 50% by the miR-200a-3p inhibitor (Figure 5B). Accordingly, the mRNA expression levels of *NOTCH1* were significantly reduced in cells transfected with miR-200a-3p mimic alone and those infected with *R. rickettsii* following delivery of miR-200a-3p mimic (Figure 5C), and opposite effects were clearly evident when the inhibitor of miR-200a-3p was used in these experiments (Figure 5D). Together, these findings suggest that miR-200a-3p directly regulates *NOTCH1* expression during *Rickettsia* infection of endothelial cells.

### 2.6. Potential Regulatory Roles of miRNAs and Target mRNAs in Signaling Networks

**Figure 6:**
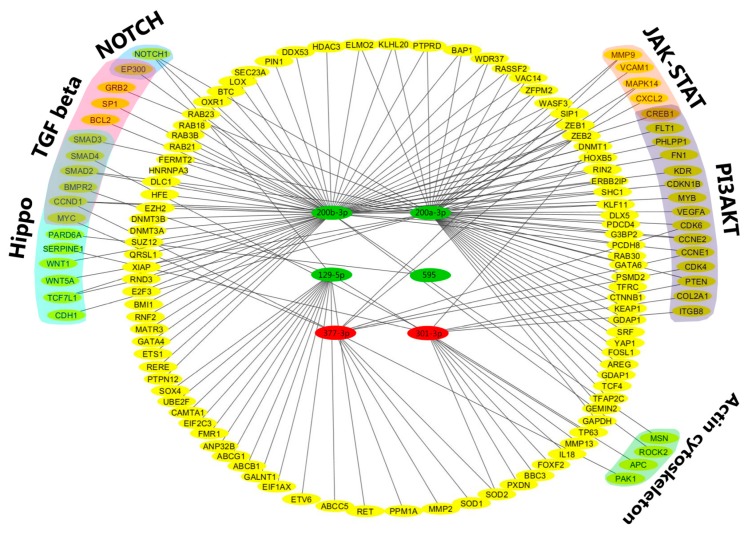
Implications of miRNA-mRNA pairs in selected pathogen-induced signaling pathways: A total of 6 miRNAs and their mRNA targets were analyzed for the potential of their involvement in signaling pathways using Cytoscape 3.2.0 (www.cytoscape.org). We have presented only a few selected pathways, which may be involved in pathogen-induced signaling. Only experimentally validated targets with strong evidence based on a combination of reporter assay, Western blot analysis, and quantitative PCR are presented. Dark green color indicates up-regulated and red color depicts down-regulated miRNAs during rickettsial infection. Yellow color represents the target genes for both up- and down-regulated miRNAs and lines indicate regulatory relationships between the miRNAs and genes. Selected genes and their involvement in respective signaling pathways are depicted in the outer circle. There are currently no validated targets with strong evidence for miR-548-3p in the databases MirTarBase [22] and DIANA-TarBase [23] used in this study.

Since miRNAs and their downstream target transcripts may cumulatively determine host cell responses and disease pathogenesis through their regulatory capacity to modulate distinct signaling pathways, we performed a bioinformatics analysis of regulatory networks of miRNAs with altered expression in *R. rickettsii*-infected host cells. The results for differentially expressed miRNAs, their validated gene targets, and the potential for involvement of these genes in selected signaling pathways are presented in Figure 6. A number of miRNA-regulated genes in infected cells were enriched for pathways involved in the organization of actin cytoskeleton and JAK-STAT or PI-3-kinase/Akt signaling, that have been demonstrated in previous studies to be activated due to rickettsial infection [24,25,26,27]. In addition, this analysis lends further support to the findings of this study illustrating the potential for regulation of *NOTCH1* by miR-200a-3p and possibly by others namely miR-200b-3p and miR-129-5p (Figure 6). Interestingly, NOTCH1 signaling intersects with several other cellular pathways, including TGF-β signaling, for which genes such as *SMAD2* and *SMAD3* appear to be down-regulated during *R. rickettsii* infection. Together, functional enrichment analysis clusters miRNAs and their target mRNAs to specialized signaling mechanisms, including NOTCH, TGF-β, and Hippo pathways, and reveals potential contributions of their regulation to the host cell responses to infection.

## 3. Discussion

The pathways of innate immunity comprise the first line of defense through which the host recognizes and responds to invading pathogens or pathogen-associated molecular patterns. Within the past decade, studies on host-pathogen interactions have increasingly intersected with the field of miRNA biology, due in particular to the important functions of miRNAs in the modulation of host cell transcriptome and as the pivotal components of host immune responses toward microorganisms. Specifically, many viral pathogens exploit the host miRNA system for their own benefit such as bypassing host immune barriers to ensure survival and replication within the host cell, while others also utilize virally-encoded miRNAs to subvert host defense mechanisms and achieve intracellular persistence. In general, viral and bacterial pathogens have now been linked to the alterations of host cellular miRNAs, which have the capacity to mitigate cell death mechanisms, cytokine signaling, and immune activation in response to infection. Pathogenic *Rickettsia* species primarily target microvascular endothelium lining the small and medium-sized vessels in their mammalian hosts and exploit cell signaling pathways to ensure entry, replication, and survival within the preferred intracellular host niche, but the possibility of altered miRNA status and the potential downstream effects of such changes on host transcriptional responses remain to be defined. In the present study, we have profiled changes in the expression of miRNAs in response to *R. rickettsii* infection of human endothelial cells. We have subsequently confirmed the alterations in the expression of important regulatory miRNAs via a complementary quantitative-PCR based approach in vitro and in a murine model of endothelial-target rickettsiosis based on *R. conorii*. Our strategic rationale for employing *R. conorii* for in vivo investigations originates from the published findings documenting that *R. conorii* infection of susceptible C3H/HeN mice closely mimics the disseminated endothelial infection as the major feature of pathogenesis and overall pathology of RMSF and MSF in humans [19,28,29], while a direct and authenticated *R. rickettsii* mouse model akin to human disease is not yet available.

Accumulating evidence reveals the importance of miRNAs in regulating key signaling and homeostasis pathways within the vasculature. As such, miRNAs have been implicated in the regulation of all aspects of endothelial biology, including identification of flow-sensitive miRNAs, control of oxidative stress, modulation of nitric oxide release, functions in vascular inflammation and angiogenesis, and mediation of intercellular communication [30]. Our global microarray-based analysis of differentially expressed cellular miRNAs illustrated a higher proportion of up-regulated miRNAs, although a few miRNAs were clearly subjected to down-regulation upon *R. rickettsii* infection. Both sets of regulated miRNAs included those that were differentially expressed at 3 or 24 h post-infection. For this first study, we decided to focus on those exhibiting consistent, most robust, and statistically significant changes in their expression. It is important to mention, however, that we also observed a significant increase of more than 10-fold in miR-146a at 24 h post-infection. MicroRNA146a plays an important role in the regulation of both innate and adaptive immunity [31,32] and has been shown to be induced in response to influenza H1N1 and H3N2, LMP1 of *Epstein-Barr* virus, and TAX protein of human T-lymphotrophic virus [33,34,35]. *R. rickettsii* infection of endothelial cells activates the transcription factor nuclear factor-kappa B (NF-κB) [36]. As an NF-κB dependent gene, miR-146a suppresses endothelial activation by inhibiting NF-κB and MAP kinase pro-inflammatory pathways and downstream EGR transcription factors through a negative feedback regulation loop [37,38]. As an endotheliotropic pathogen similar to *Rickettsia* species, *Orientia tsutsugamushi* has also been demonstrated to induce miR-146a in macrophages in a dose-dependent manner [39].

Although miRNA profiling provides a broad overview of the presence and regulation of miRNAs and may reflect their potential biological roles in normal cellular function and disease, it is critically necessary to verify the resultant data by miRNA-specific approaches such as quantitative analysis employing stem loop specific reverse transcription primers and/or miRNA-specific primer(s) for PCR. A total of seven miRNAs deregulated in *R. rickettsii*-infected endothelial cells were thus further confirmed in this study, of which four (miR-129-5p, miR-200a-3p, miR-200b-3p, and miR-595) were up-regulated and another three (miR-301b-3p, miR-548a-3p, and miR-377-3p) were down-regulated. Considering that miRNA phenotypes observed in vitro might differ from their regulation in vivo due to differences in biogenesis, further assessment of four of these miRNAs (two up-regulated and two down-regulated) in the lungs as the major target organs of mice infected with *R. conorii* revealed an identical pattern of regulation, suggesting potential for their involvement in host responses to *Rickettsia* infection. It is important to note that *R. conorii* used for in vivo infection is antigenically and phylogenetically similar to *R. rickettsii* and causes disease in susceptible mice akin to spotted fever in humans [3,19,28]. Also, modulation of host miRNAs by viruses and facultative intracellular bacterial pathogens has been the focus of a number of studies, but there is only limited information on this aspect in the biomedical literature for obligate intracellular bacteria. Thus, miRNAs identified in our study as possible post-transcriptional controllers of host-*Rickettsia* interactions are novel and warrant further investigation, since not much is known regarding their contributions to antibacterial immune responses or host cell death mechanisms.

Excessive reactive oxygen species, especially intracellular peroxides including superoxide anion, have been documented to play important roles in the pathogenesis of *R. rickettsii* infection [40]. It has also been suggested that as a consequence of generation of superoxide radicals by infected endothelium, the enzymatic activity of superoxide dismutase is also increased, despite evidence for compromised activities of other anti-oxidant enzymes such as catalase and glutathione peroxidase [41,42]. Superoxide dismutases (SODs) represent a major antioxidant defense system in mammals and require a catalytic metal (Cu or Mn) for their activation. The isozymes of SOD include cytoplasmic Cu/ZnSOD (SOD1), mitochondrial MnSOD (SOD2), and the extracellular Cu/ZnSOD (SOD3). Thus, increased mRNA expression of *SOD1* and *SOD2* observed in this study is in agreement with previous findings of increased enzymatic activity during *R. rickettsii* infection of human endothelial cells [41,43]. Intriguingly, *SOD1* and *SOD2* are validated targets of miR-377-3p and miR-377-3p/miR-301b-3p, respectively, and both of these miRNAs are down-regulated in our system, suggesting the to-be-tested hypothesis for potential correlation between changes in the transcription of inducible *SOD* isoforms and selective host miRNAs due to rickettsial infection.

Notch receptors constitute a highly conserved signaling pathway crucial for the development and regulation of innate immunity and inflammation in health and disease. Simplistically, the fundamental paradigm of Notch signaling involves proteolytic cleavage to release Notch intracellular domain, which then translocates to the nucleus to associate with CSL (suppressor of hairlessness) and co-activators to induce transcription of target genes. NOTCH1 is broadly expressed in many human tissues, including vascular endothelial cells [44]. In endothelial cells, NOTCH1 mediates the proliferation and migration induced by vascular endothelial growth factor and its antiapoptotic and angiogenic effects [45]. Lymphatic endothelial cells infected with Kaposi sarcoma-associated herpesvirus exhibit induction of Notch receptor signaling [46], and *Mycobacterium bovis* BCG has been demonstrated to activate NOTCH1 expression and signaling in macrophages [47]. Evidence also suggests that a tandem repeat protein TRP120 of obligately intracellular *Ehrlichia chaffeensis* activates canonical NOTCH1 signaling to evade host immune response and promote bacterial survival [48]. Our data suggests an inhibitory cross-talk between miR-200a-3p and *NOTCH1* expression and a potentially important role for this interrelationship in rickettsial biology, because NOTCH1 signaling interacts with several other cellular pathways, transcription, and growth factors regulating various mechanisms including determination of differentiation and cell fate, endothelial proliferation, and angiogenesis in the vasculature [49]. It is interesting to note that *SMAD2* and *SMAD3*, known to have distinct and non-overlapping roles in TGF-β signaling are also down-regulated in the context of *R. rickettsii* infection of endothelial cells. Considering that the NOTCH pathway can be easily manipulated with inhibitors of γ-secretase and metalloprotease, further investigations to elucidate the roles of miR200a-3p, miR-200b-3p, miR-129-5p and possibly other miRNAs that could be mechanistically linked with NOTCH and other signal transduction pathways should allow for their exploitation as a new therapeutic strategy.

## 4. Materials and Methods

### 4.1. Endothelial Cell Culture, Infection, and Transfection

*Rickettsia rickettsii* (Strain Sheila Smith) was propagated in Vero cells to prepare partially purified stocks as described previously [50]. The infectivity titers of these stocks to be used for in vitro infection of human dermal microvascular endothelial cells (HMECs) were determined by quantitative PCR and plaque formation assay [50,51] and the stocks were kept frozen as aliquots at −80 °C. HMECs were cultured in MCDB 131 medium (Caisson’s Laboratories, Smithfield, UT, USA), supplemented with FBS (10% *v*/*v*; Aleken Biologicals, Texarkana, TX, USA), epidermal growth factor (Sigma, St. Louis, MO, USA, 10 ng/mL), l-glutamine (10 mM, Thermo Fisher Scientific, Waltham, MA, USA), and hydrocortisone (1 μg/mL, Sigma). HMECs were infected with *R. rickettsii* at an MOI of 1:5 according to established procedures, because this dose of infection consistently results in activation of host cell signaling mechanisms and downstream responses in a variety of endothelial cells [50]. At 3 h (to allow for adhesion, entry, and establishment of intracellular infection) and 24 h (to allow for at least two replication cycles of intracellular rickettsiae) post-infection, culture medium was removed by aspiration and the cells were lysed in TRI-reagent^®^ (Molecular Research Center, Inc., Cincinnati, OH, USA). In all experiments, the viability of both uninfected and *R. rickettsii*-infected HMECs was ascertained microscopically. The mimic and inhibitor for miRNA-200a-3p along with the negative controls were purchased from Applied Biosystems and transfected into HMECs in culture using Lipofectamine^®^ RNAiMAX according to the manufacturer’s recommendations for 48 h prior to infection with *R. rickettsii*. The negative controls for miRNA mimics and inhibitors are random nucleotide sequences that have been extensively tested in human cell lines and validated to produce no identifiable effects on known miRNA functions.

### 4.2. RNA Preparation

Total RNA was extracted from *R. rickettsii-*infected and uninfected HMECs according to the standard TRI-reagent^®^ protocol in accordance with the manufacturer’s recommendations. Briefly, cell lysates in TRI-reagent^®^ were gently mixed with chloroform (5:1 *v*/*v*) and aqueous phase containing the RNA was separated into a sterile tube. RNA was then precipitated using 0.7 volumes of isopropanol and pelleted by centrifugation, washed with 70% ethanol, and allowed to dry at room air and temperature prior to solubilization in nuclease-free water. RNA preparations thus obtained were subjected to treatment with DNase I to remove any contaminating DNA and quantitated using a MultiSkan™ Go Spectrophotometer (Thermo Fisher Scientific, Waltham, MA, USA). The RNA quality was next assessed by visualization of the bands for 18S and 28S RNA on an Agilent Bioanalyzer 2100 (Agilent Technologies, Santa Clara, CA, USA). The electropherogram for each sample was used to determine the 28S:18S ratio and the RNA Integrity Number [52]. RNA preparations with a RIN number ≥9.0 were used in the experiments.

### 4.3. MicroRNA Expression Analysis by Affymetrix GeneChip^®^ Microarray

Total RNA (500 ng) containing small/miRNAs was labeled for microarray analysis using Flashtag™ HSR RNA labeling kit (Genisphere, PA, USA) according to the manufacturer’s instructions. The labeled products were hybridized to a GeneChip^®^ miRNA 2.0 Array (Affymetrix, Santa Clara, CA, USA) at 48 °C and 60 rpm for 16 h. The chips were washed and stained in a GeneChip Fluidics Station 450 (Fluidics script FS450_0003) and fluorescence detected with an Affymetrix-7G Gene Array scanner using the Affymetrix GeneChip Command Console software (AGCC1.1). Three separate replicates were included for each experimental condition. The image data were analyzed with the miRNA QC Tool software for quality control. The miRNA expression changes were identified using Partek Genomics Suite (Partek, Chesterfield, MO, USA) following the default miRNA expression workflow. The resulting values were then filtered for *p*-values of ≥0.05 and a relative fold-change between −2 and 2.

### 4.4. Quantitative Real Time PCR for Analysis of miRNA and mRNA Expression

TaqMan^®^ two-step RT-PCR assays containing primers for both miRNA-specific reverse transcription and quantitative PCR were obtained from Applied Biosystems. One microgram of total RNA each was reverse transcribed using the TaqMan MicroRNA cDNA synthesis kit (Applied Biosystems, Foster city, CA, USA) using miRNA-specific primers as well as oligo (dT) primers for the analysis of 18S and GAPDH expression. A negative control lacking RT was included in all PCR reactions. The expression of miRNAs was analyzed by real-time PCR using the TaqMan^®^ assay specific for each microRNA (Applied Biosystems). Amplification of 18S RNA and GAPDH was employed as an endogenous control and used to normalize miRNA and mRNA expression respectively among sample sets [53,54]. The mean *C*_T_ values for experimental (infected) samples were normalized to the baseline controls (uninfected), which were assigned a value of 1, and the relative expression was calculated by comparative *C*_T_ (^ΔΔ^*C*_T_) method as described in [55]. Specifically, we measured the amplification of the target and housekeeping genes in infected and control samples and the *C*_T_ values for target genes were normalized to that of the housekeeping species using the StepOne™ Plus software version 2.3 (Applied Biosystems, Foster City, CA, USA). We next determined relative quantitation by comparing normalized target quantity in each experimental (infected) sample to the normalized target quantity in uninfected controls. The data sets were calculated as the mean ± SE from a minimum of three independent experiments performed as technical triplicates.

### 4.5. Identification of Target Genes

The target genes regulated by differentially expressed miRNAs were identified by computational analysis of two databases listing experimentally validated miRNA-target mRNA interactions, namely MirTarBase [22] and DIANA-TarBase [23]. The candidate genes thus identified as potential downstream targets of selected miRNAs were screened subsequently through measurement of their expression in *R. rickettsii*-infected HMECs and corresponding uninfected controls. The primers utilized in the study are listed in Table S1.

### 4.6. In Vivo Model of Infection

All animal experiments were performed in accordance with the protocol approved by the Institutional Animal Care and Use Committee. C3H/HeN mice (Harlan Sprague Dawley, Indianapolis, IN, USA) were intravenously infected with 2.25 × 10^5^ pfu of *R. conorii* per animal. Control animals received injection of saline. On day 3 post-infection (the earliest time at which the mice display the sign of disease), mice were euthanized and lungs were removed aseptically for isolation of total RNA and analysis by q-RT-PCR using miRNA-specific Taqman assays.

### 4.7. Statistical Analysis

All experiments were performed at least three times with technical triplicates and the data are presented as the mean ± Standard Error (SE). Statistical analysis for differentially expressed miRNAs in *R. rickettsii-*infected and uninfected groups was performed by one/two way ANOVA with Dunnett’s post-test using GraphPad Prism 4.00 (GraphPad Software Inc., La Jolla, CA, USA). The *p* value for statistical significance among data sets was set at ≤0.05.

## 5. Conclusions

To summarize, the findings of this study present the first evidence for a significantly altered “miRNA expression pattern” during rickettsial infection of host cells and provide the foundation for further in-depth investigations of the contributions of each specific miRNA in the mechanisms of gene regulation relevant to host-pathogen interactions and vascular cell biology. Ongoing investigations on selected miRNA-governed regulation of target genes are expected to provide new perspectives on the host response and pathogenesis mechanisms, which would help design new therapeutic and/or diagnostic strategies for human rickettsioses.

## Figures and Tables

**Table 1:** List of human miRNAs significantly up and down regulated in human dermal microvascular endothelial cells (HMECs) upon infection with *R. rickettsii* strain Sheila Smith.

miRNA Name	Consensus Sequence	Fold Change	
**Up Regulated miRNAs**		**3 h**	**24 h**
miR-129-5p	CUUUUUGCGGUCUGGGCUUGC	19.8	9.5
miR-200a-3p	UAACACUGUCUGGUAACGAUGU	15.9	8.8
miR-297	AUGUAUGUGUGCAUGUGCAUG	15.6	18.6
miR-200b-3p	UAAUACUGCCUGGUAAUGAUGA	14.7	10.2
miR-595	GAAGUGUGCCGUGGUGUGUCU	10.4	9.3
miR-574-5p	UGAGUGUGUGUGUGUGAGUGUGU	7.2	8.6
miR-647	GUGGCUGCACUCACUUCCUUC	4.1	5.0
miR-615-3p	UCCGAGCCUGGGUCUCCCUCUU	3.9	4.3
miR-1224-5p	GUGAGGACUCGGGAGGUGG	3.5	4.2
miR-1238-3p	CUUCCUCGUCUGUCUGCCCC	2.2	2.2
miR-196a-5p	UAGGUAGUUUCAUGUUGUUGGG	3.1	-
miR-616-3p	AGUCAUUGGAGGGUUUGAGCAG	3.9	-
miR-760	CGGCUCUGGGUCUGUGGGGA	3.7	-
miR-1275	GUGGGGGAGAGGCUGUC	2.1	-
miR-146a-5p	UGAGAACUGAAUUCCAUGGGUU	-	13.7
miR-631	AGACCUGGCCCAGACCUCAGC	-	12.6
miR-661	UGCCUGGGUCUCUGGCCUGCGCGU	-	7.7
miR-766-3p	ACUCCAGCCCCACAGCCUCAGC	-	4.6
miR-1273a	GGGCGACAAAGCAAGACUCUUUCUU	-	2.8
miR-1202	GUGCCAGCUGCAGUGGGGGAG	-	2.7
miR-943	CUGACUGUUGCCGUCCUCCAG	-	2.7
miR-1250-5p	ACGGUGCUGGAUGUGGCCUUU	-	2.6
miR-1229-3p	CUCUCACCACUGCCCUCCCACAG	-	2.5
**Down Regulated miRNAs**			
miR-301b-3p	CAGUGCAAUGAUAUUGUCAAAGC	−3.3	−2.8
miR-548a-3p	CAAAACUGGCAAUUACUUUUGC	−3.1	−3.1
miR-377-3p	AUCACACAAAGGCAACUUUUGU	−2.1	−3.2
miR-602	GACACGGGCGACAGCUGCGGCCC	−2.1	-
miR-802	CAGUAACAAAGAUUCAUCCUUGU	−2.2	-
miR-376b-3p	AUCAUAGAGGAAAAUCCAUGUU	-	−2.9
miR-216b-5p	AAAUCUCUGCAGGCAAAUGUGA	-	−2.4
miR-216a-5p	UAAUCUCAGCUGGCAACUGUGA	-	−2.4
miR-410-3p	AAUAUAACACAGAUGGCCUGU	-	−2.3
miR-29b-3p	UAGCACCAUUUGAAAUCAGUGUU	-	−2.1

‘-’ indicates fold change <2 and/or *p* > 0.05.

**Table 2:** List of potentially relevant, validated target genes for miRNAs regulated by *R. rickettsia*.

miRNAs	Validated Target Genes (miRTarBase and DIANA-TarBase)
miR-129-5p	*CAMTA1*, *SOX4*, *NOTCH1*, *UBE2F*, *CDK6*, *BMPR2*, *GALNT1*, *FMR1*
miR-200a-3p	*NOTCH1*, *CDK6*, *ZEB1*, *ZEB2*, *SMAD2*, *SMAD3*, *RIN2*, *VCAM1*, *KEAP1*
miR-200b-3p	*ZEB1*, *ZEB2 SIP1*, *SMAD2*, *SMAD3*, *VEGFA*, *GATA4*, *KDR*, *XIAP*, *RIN2*
miR-595	*CALM1*, *TNIP2*, *PARD6A*, *ULK1*, *ACP1*, *SASH3*, *RAB10*, *HINT1*
miR-548a-3p	*DSC2*, *FGF-7*, *CALM1*, *ILDR1*, *PRLR*, *TRIM13*, *PARP15*, *SOX4*
miR-377-3p	*PAK1*, *SOD1*, *SOD2*, *SMAD5*, *PKD1*, *IGF2R*, *FKBP5*, *CARD8*
miR-301b-3p	*OXA1L*, *FGFR1*, *SMAD4*, *LDLR*, *TGFBR2*, *SOX4*, *EDN1*, *SOD2*

## Supplementary Materials

Supplementary materials can be found at http://www.mdpi.com/1422-0067/18/7/1471/s1.
