# Supplementary material for: MicroRNA Signature of Human Microvascular Endothelium Infected with Rickettsia rickettsii
Source: Int J Mol Sci. 2017 Jul 9;18(7):1471. doi: 10.3390/ijms18071471 (PMC5535962; doi:10.3390/ijms18071471)
Supplement: Supplementary file 1 [file ijms-18-01471-s001.pdf]

MicroRNA Signature of Human Microvascular Endothelium Infected with *Rickettsia rickettsii*

Abha Sahni, Hema P. Narra, Jignesh Patel and Sanjeev K. Sahni

Table S1. List of primers used in this study.

Gene	Orientation	Primer Sequence (5′-3′)
GAPDH	Forward	CTGGTAAAGTGGATATTGTTGCCAT
	Reverse	TGGAATCATATTGGAACATGTAAACC
NOTCH1	Forward	CGCTGACGGAGTACAAGTG
	Reverse	GTAGGAGCCGACCTCGTTG
SMAD2	Forward	TCATAGCTTGGATTTACAGCCAG
	Reverse	TTCTACCGTGGCATTTCGGTT
SMAD3	Forward	GCGTGCGGCTCTACTACATC
	Reverse	GCACATTCGGGTCAACTGGTA
RIN2	Forward	GCCTGGAACCCGCTGAA
	Reverse	CGGAATAGCCACCATCCTTGT
SOD1	Forward	TGGGCCAAAGGATGAAGAGA
	Reverse	TAGACACATCGGCCACACCAT
SOD2	Forward	CAGACCTGCCTTACGACTATGG
	Reverse	CTCGGTGGCGTTGAGATTGTT
18S	Forward	GTAACCCGTTGAACCCCATT
	Reverse	CGCTACTACCGATTGGATGG
